# Points from Hospital Reports

**Published:** 1924-04

**Authors:** 


### POINTS FROM HOSPITAL REPORTS.

### Aberdeen Royal Infirmary.

The Directorate of this hospital of 350 beds are to be congratulated on a fine year's work, and on the high efficiency of their management, which is in evidence in whatever direction we turn. The salient features in the record of 1923 are:—In-patients more numerous than in any previous year; beds increased in number to meet the demand; expenditure well under control in all departments; income well maintained and adequate to the demands upon it, with a margin; truly an enviable condition. The Report and audit of accounts bear date January 30, 1924. Among the interesting points that have attracted our notice the following may be mentioned:—A donation of £277 from the Hon. Medical Staff from the Ministry of Pensions Grant for the treatment of Discharged Disabled Sailors and Soldiers; the strikingly small amount (£315) of medical salaries, not a tithe of what obtains in London general hospitals of similar size; the fact that no fewer than 234 applications were received during the year from women desiring to enter for training in general nursing; the reduction in the cost per occupied bed from £104 to £99; and the exclusion from revenue available to meet expenditure of all legacies exceeding £200 in individual amount, which are carried to a fund for special or extraordinary purposes. The Report contains valuable tabular statements for ten years of Income, Expenditure, Averages, etc. We suggest that it would be well to add a column to the last-mentioned for "beds available." There is no index—a defect easily remedied.

### Sunderland Royal Infirmary.

This Report deals with the work and finance of the Infirmary itself, the Children's Hospital, and the Heatherdene Convalescent Home, for 1923. In each establishment and in every department the work done was greater than in 1922; and it is noteworthy that 95 per cent. of the beds in the Infirmary were constantly occupied—a high proportion. The total receipts were £43,259 and payments £46,083. There was therefore a deficiency of £2,824, which is within a few hundred pounds of the amount by which donations to the Infirmary fell below those of the preceding year. We mention this merely as a fact, and not as suggesting cause and effect. The outstanding financial feature, especially in view of the great prevalence of unemployment, is the very large sum of £20,692 received from workpeople's contributions—nearly 45 per cent of the total expenditure of the year. Furthermore, it is some £950 in advance of the preceding year's figure, which goes to show that it is progressive, not exceptional. It is highly satisfactory and is a source of great financial stability. The accounts do not include a balance-sheet, and we are not sure whether they are accounts of receipts and payments or of income and expenditure. They are headed with the former title, and yet are specially labelled as having been "brought into line" with the uniform system, while they are referred to in the Report itself as the latter. The point is interesting in relation to the despatch with which the books were closed and audited (January 31), a date easier to reconcile with an account of receipts and payments than with one of income and expenditure.

### Blackburn and East Lancashire Royal Infirmary.

This general hospital of 152 beds is flourishing. Notwithstanding trade depression, the authorities have been able to meet the expenditure of 1923, while leaving the "open" legacies unencroached upon and available for capital expenditure. We congratulate them especially on the large and progressive amount (no less than £12,301, compared with a total ordinary expenditure of £23,190—more than half) furnished by workpeople's funds. It is a great record in such hard times, and is an eloquent testimony to the high esteem in which the hospital is held by the people of East Lancashire. The work of the year—both in-patient and out-patient—was greater than ever. We presume the figures given in the Medical Report are correct—total beds available 152, average number occupied daily 156.12; and, if so, it is small wonder that the Report speaks of the crowded condition of the institution. This leads up to the question of extensions, which are not only in contemplation, but for the main part of which the contract has already been placed. This is a new wing, which is to be Blackburn's War Memorial, and is estimated to cost £75,000. It will provide ninety additional beds, two operating rooms with their adjuncts, and a new block of administrative offices. Before this wing can be occupied a new centralised kitchen department, a laundry and boiler house are essential. There is also foreshadowed a new out-patient department, clinical and pathological laboratories and a nurses' home. The whole is a formidable undertaking, but the authorities face it with courage and resource. We earnestly wish them full success and shall watch their progress with great interest. The Report is without an index, and we had to read to the eighteenth page before we could discover the number of beds.

### The Clayton Hospital, Wakefield.

The people of Wakefield, the capital of the West Riding of Yorkshire, have reason to be proud of their hospital. Judging from the photograph on page 2 of the Report, it appears to be a picturesque building, situated in charming grounds; and that the second of these appearances, at any rate, is no illusion is proved by the following pleasing paragraph which we quote from the Report:—"The Board are pleased to put on record the great satisfaction they feel at the appearance of the Hospital Grounds, which are kept in splendid condition and reflect much credit on the gardener (F. Walker)." But its natural advantages, with all the added charm imparted by the attentions of Mr. Walker, by no means exhaust its merits. Indeed, its external attractiveness is but an indication of the quality of its internal economy, practical evidence of which is afforded by the following facts:—Work well maintained; income within £1 6s. 10d. of equality with expenditure, one-third of it derived from workpeople's contributions, and nearly another third from investments; annual subscriptions increased by £359; expenditure substantially reduced. A new nurses' home is on the eve of being built, as part of Wakefield's War Memorial. It is to cost £10,000, of which £7,000 is in hand. The Report is an excellent compilation, containing in a concise form much interesting and useful information. The hospital was founded in 1854 and contains 108 beds.

### Staffordshire County Asylums.

Several matters of extreme interest in asylum life and administration have been raised in the recently-issued reports of these institutions. In passing it may be noted that they indicate how alive the various committees and medical superintendents are to the necessity of progressive amelioration of the conditions of life in the asylums which they administer. Consider the state of affairs at Cheddleton. The Committee "regret to have to confess that the fabric of the building has further deteriorated during the past year, and that the amounts expended from the County Rate grants have been insufficient to keep pace with decay"; and they draw a lamentable picture of the more urgent needs. The Nurses' Home, although opened in 1914, "has never been painted, nor have the public rooms been furnished." This may have some connection with the statement which appears later in the Report that there has been difficulty in obtaining nurses. Dr. B. H. Shaw comments on the undue proportion of tuberculous patients at the Stafford Mental Hospital, although he points out that there has been a further drop in tuberculous mortality. It was 14.8 per 1,000 resident which, "while it leaves much to be desired when compared with a mortality of .72 for the general population of Staffordshire, is a very considerable improvement on the pre-war average of 36.67." In order to see what steps could be taken to reduce the liability to infection an exhaustive bacteriological investigation was undertaken to determine the effect of different methods of floor treatment on the microbic content of dust from various wards so far as human contamination by pathogenic organisms was concerned. The results show clearly that the installation of vacuum-cleaning methods would do very much to prevent infection. "The evil effects of sweeping and polishing floors of wards constantly occupied by large numbers of patients were clearly shown. For instance, for every 100 organisms deposited in ten minutes in a certain small area when a ward floor was undisturbed for three hours, 1,211 were deposited fifteen minutes after sweeping, 1,439 after sweeping with damp sawdust and tea leaves, 228 after polishing, and only 86 after the use of a vacuum dust extractor."

